# Neuronal Calcium Sensor-1 Binds the D2 Dopamine Receptor and G-protein-coupled Receptor Kinase 1 (GRK1) Peptides Using Different Modes of Interactions*

**DOI:** 10.1074/jbc.M114.627059

**Published:** 2015-05-15

**Authors:** Sravan Pandalaneni, Vijaykumar Karuppiah, Muhammad Saleem, Lee P. Haynes, Robert D. Burgoyne, Olga Mayans, Jeremy P. Derrick, Lu-Yun Lian

**Affiliations:** From the ‡ NMR Centre for Structural Biology, Institute of Integrative Biology, and; ‖ Institute of Integrative Biology, University of Liverpool, Liverpool L69 7ZB,; the § Faculty of Life Sciences, University of Manchester, Manchester M13 9PT, and; the ¶ Physiological Laboratory, Department of Cellular and Molecular Physiology, Institute of Translational Medicine, University of Liverpool, Liverpool L37 4BY, United Kingdom

**Keywords:** calcium, dopamine, G protein-coupled receptor (GPCR), receptor internalization, x-ray crystallography, D2 dopamine receptor, GRK1, neuronal calcium sensor-1

## Abstract

Neuronal calcium sensor-1 (NCS-1) is the primordial member of the neuronal calcium sensor family of EF-hand Ca^2+^-binding proteins. It interacts with both the G-protein-coupled receptor (GPCR) dopamine D2 receptor (D2R), regulating its internalization and surface expression, and the cognate kinases GRK1 and GRK2. Determination of the crystal structures of Ca^2+^/NCS-1 alone and in complex with peptides derived from D2R and GRK1 reveals that the differential recognition is facilitated by the conformational flexibility of the C-lobe-binding site. We find that two copies of the D2R peptide bind within the hydrophobic crevice on Ca^2+^/NCS-1, but only one copy of the GRK1 peptide binds. The different binding modes are made possible by the C-lobe-binding site of NCS-1, which adopts alternative conformations in each complex. C-terminal residues Ser-178–Val-190 act in concert with the flexible EF3/EF4 loop region to effectively form different peptide-binding sites. In the Ca^2+^/NCS-1·D2R peptide complex, the C-terminal region adopts a 3_10_ helix-turn-3_10_ helix, whereas in the GRK1 peptide complex it forms an α-helix. Removal of Ser-178–Val-190 generated a C-terminal truncation mutant that formed a dimer, indicating that the NCS-1 C-terminal region prevents NCS-1 oligomerization. We propose that the flexible nature of the C-terminal region is essential to allow it to modulate its protein-binding sites and adapt its conformation to accommodate both ligands. This appears to be driven by the variability of the conformation of the C-lobe-binding site, which has ramifications for the target specificity and diversity of NCS-1.

## Teaser

**Background:** Neuronal calcium sensor-1 (NCS-1) is a Ca^2+^-binding protein that interacts with neuronal proteins.

**Results:** NCS-1 binds the dopamine D2 receptor and its cognate kinase in different ways.

**Conclusion:** The C terminus and EF3-EF4 linker of NCS-1 modulate its binding to these target proteins.

**Significance:** The specificity and diversity of NCS family proteins are determined by the C-lobe-binding site conformation.

## Introduction

Ca^2+^ is known to trigger the release of neurotransmitters in synapses. The transmission of information through the central nervous system, therefore, relies on changes in intracellular free Ca^2+^ concentration. It is well established that the response to changes in Ca^2+^ concentration that results in neurotransmitter release is mediated by the Ca^2+^-binding protein synaptotagmin (1). Many other aspects of neuronal function are modified through the actions of other Ca^2+^-binding proteins. For example, changes in synaptic plasticity and gene expression in neurons can involve the Ca^2+^-binding protein calmodulin (2, 3). Other aspects of neuronal function are regulated by the neuronal calcium sensor (NCS)^5^ family of EF-hand-containing proteins (4). NCS proteins have a much higher affinity for Ca^2+^ than calmodulin; this fact means that NCS proteins bind Ca^2+^ following much smaller increases in Ca^2+^ concentration above resting levels. In addition, the higher affinity of NCS proteins for Ca^2+^ limits the dynamic range over which these proteins can respond to changes in intracellular Ca^2+^ concentration (5).

NCS-1, a member of the NCS family, has been reported to be involved in many important physiological functions (6), ranging from the regulation of neurotransmitter release (7, 8) to neuronal development (9, 10), and learning (11, 12). The target proteins that interact directly with NCS-1 to cause some of these physiological effects (6, 13, 14) include phosphatidylinositol 4-kinase (PI4K) IIIβ (15, 16), ARF1 (15, 17), the dopamine D2 receptor (18), and G-protein-coupled receptor (GPCR) kinases GRK1 (19) and GRK2 (18, 20).

The GPCR D2R is the primary isoform found in the brain; its physiological relevance is illustrated by the fact that it is the target for all known effective antipsychotic drugs (21). The activity of GPCRs can be terminated by the phosphorylation of their activated states by the GPCR kinase family of proteins (22). NCS-1 binds to both the D2R and its cognate kinases GRK1 and GRK2; for example, an NCS-1·D2R·GRK2 ternary complex has been detected (18), with GRK2 promoting the desensitization of the D2R. NCS-1 binds D2R at the short 16-residue intracellular C-terminal region (18, 23). NCS-1 interaction with and regulation of D2R is important, because this forms the link between the overexpression of NCS-1 with spatial memory acquisition (12) and explains why NCS-1 is required for an adaptive response to dopaminergic agonists in substantia nigra neurons (20). Significantly, NCS-1 is up-regulated in patients with bipolar disorder or schizophrenia (24) and in response to anti-psychotic drugs (25). Knowledge of the molecular basis for the recognition of D2R and GPCR kinase by NCS-1 could contribute to the development of drugs that are specific for this signaling pathway.

Here, we report the determination of the crystal structures of *Rattus norvegicus* NCS-1 in complex with peptides derived from D2R and GRK1, as well as the structures of NCS-1 alone and in a C-terminal truncated form. In the complex structures, each NCS-1 molecule binds two copies of the D2R peptide but only one GRK1 peptide in overlapping, although not identical, binding sites. These structures show that NCS-1 could simultaneously bind D2R and GRK1 peptides and thus act as a small scaffold protein. Significant conformational changes are observed in the C-terminal region of NCS-1 in both complexes to facilitate this function. We also show that removal of this section of the C terminus leads to the formation of an NCS-1 dimer. Based on the crystal structures determined here, we proposed an induced-fit mechanism for NCS-1 recognition of its targets, which requires flexibility of both the C terminus and the EF3/EF4 linker regions.

## Experimental Procedures

### 

#### 

##### Plasmids

A mutant construct NCS-1(1–177), where the last 13 residues were removed, was generated by introducing a stop codon after proline 177 using an antisense strand 5′-CGGATCCGGTACCTTACTAGGGGTCGGCCTTGGAGCC-3′.

##### Peptide Synthesis

The D2 receptor peptide (D2R peptide) used here (NIEFRKAFLKILHSR) corresponds to residues 430–443 of the human D2 receptor, with the exception that the Ser replaced Cys in the original sequence (UniProt P14416.2), and the terminal Arg was added to improve solubility. The N terminus of GRK1 (referred to as GRK1 peptide) corresponds to residues 1–25 (MDFGSLETVVANSAFIAARGSFDGS) of GRK1 (UniProt Q15835). The synthetic peptides were purchased from GenicBio, China, and delivered >95% pure.

##### Protein Purification

*R. norvegicus* NCS-1 full-length and NCS-1Δ CT were expressed in *Escherichia coli* BL21 (DE3) (Novagen) and purified as described previously (23).

##### Multiangle Laser Light Scattering (MALLS)

Measurements were performed on a Dionex BioLC HPLC connected to an 18-angle light scattering detector and a differential refractometer (DAWN HELEOS-II and OPTILab rEX, Wyatt). A Superdex 75 10/300 GL column (GE Healthcare) was used in 20 mm Tris-HCl, 150 mm NaCl, 1 mm CaCl_2_, pH 7.5, at a flow rate of 0.75 ml/min. Sample volumes of 1 ml were injected at a concentration of 1.5 mg/ml. Samples eluting from the column passed through an in-line DAWN HELEOS-II laser photometer (λ = 658 nm) and an OPTILab rEX refractometer with a QELS dynamic light scattering attachment. Light scattering intensity and eluant refractive index (concentration) were analyzed using ASTRA version 5.3.4.13 software to give a weight-averaged molecular mass. To determine the detector delay volumes and normalization coefficients for the MALLS detector, a BSA sample (Sigma A-8531) was used as reference.

##### Isothermal Titration Calorimetry

Isothermal titration calorimetry (ITC) experiments were performed using a MicroCal ITC200 instrument, and by titrating Ca^2+^/NCS-1 into the D2R peptide. NCS-1 stocks at 1 mm were prepared by buffer exchange using a PD10 column (GE Healthcare), equilibrated in 50 mm Tris-HCl, 50 mm NaCl, 5 mm CaCl_2_, pH 7.5. The D2R peptide was dissolved in water to its solubility limit of 1 mm; the pH was adjusted to 7.5, and CaCl_2_ was added to achieve a final concentration of 5 mm CaCl_2._ The D2R peptide sample in the ITC cell was prepared from this stock by dilution using the NCS-1 buffer (50 mm Tris-HCl, 50 mm NaCl, 5 mm CaCl_2_, pH 7.5). Experiments were carried out using 200 μl of 100 μm D2R peptide in the cell and 60 μl of 1 mm NCS-1 in the syringe at 25 °C. The first injection was 0.5 μl, and these data were discarded. The subsequent 20 injections of 2 μl were made with 180-s spacing to allow the baseline to return after each injection. The experiments were performed in triplicate.

##### NMR Spectroscopy

NCS-1 was prepared in 50 mm Tris-HCl buffer, pH 6.8, in the presence of 5 mm MgCl_2_ and 5 mm CaCl_2_. NMR spectra were recorded at 27 °C on Bruker DRX 800 and 600 MHz Avance II spectrometers equipped with CryoProbes. Data were processed using the Bruker software TopSpin and analyzed using CCPN software (26). Sequence-specific backbones were obtained using the HNCA, HN(CO)CA, HNCO, HN(CA)CO, CBCA(CO)NH, CBCANH, HBHA(CO)NH, and HCCH-TOCSY experiments.

##### Crystallization

Purified proteins were equilibrated against 20 mm Tris-HCl, pH 7.5, and concentrated to 1 mm final protein concentration before initiating crystallization trials. For crystallization of the peptide complexes, a 2-fold excess of peptide was added to the protein solution. Crystals were grown at 20 °C using the sitting drop vapor diffusion method. NCS-1 was crystallized in 0.1 m sodium cacodylate, pH 6.5, 0.2 m sodium acetate, and 30% (w/v) PEG 8000, similar to the conditions previously reported (27). The crystallization conditions for NCS-1·D2R complex were 150 mm Tris-HCl, pH 8.0, 8% (v/v) ethylene glycol, 20% (w/v) PEG 5000; the crystal was cryo-protected by addition of glycerol into the crystal growth medium to give 20% (v/v). The crystallization conditions for the NCS-1·GRK1 peptide complex were 0.12 m alcohols (1,6-hexanediol; 1-butanol; 1-propanediol (racemic); 2-propanol; 1,4-butanediol; 1,3-propanediol), 0.1 m Buffer 2 (sodium HEPES; MOPS acid, pH 7.5), 30% PEGMME 550 and PEG 20000; the crystal was cryo-protected using the crystal growth medium plus 20% (v/v) glycerol. The crystallization conditions for NCS-1Δ CT were 0.1 m MES, pH 6.0, 200 mm NaCl, 16% (w/v) PEG 6000; the crystal was cryo-protected using the crystal growth medium plus 25% (w/v) glycerol.

##### Structure Determination and Refinement

Data statistics and model parameters for all four structures are listed in Table 1. Data for the NCS-1·D2R and NCS-1·GRK1 complexes were processed using XDS (28), implemented from within the xia2 system for automated data reduction (29). Space group assignment was assisted using POINTLESS (30).

**TABLE 1:** **Data collection and refinement statistics**

	Apoprotein	D2R peptide complex	NCS1-CT	GRK1 peptide complex
Space group	*P2*_1_	*P 4*_1_*2*_1_*2*	*P 2*_1_*2*_1_*2*_1_	*P 2*_1_
Unit cell parameters	*a* = 53.91 Å, *b* = 55.49 Å, *c* = 77.36 Å, β = 94.4^o^	*a* = 44.67 Å, *b* = 44.67 Å, *c* = 205.52 Å	*a* = 72.68 Å, *b* = 88.80 Å, *c* = 100.67 Å	*a* = 40.69 Å, b = 93.69 Å, *c* = 55.71 Å, β = 92.3^o^
X-ray source and wavelength ( Å)	DLS*^a^* I03 (0.9763)	DLS I04-1 (0.9173)	DLS*^a^* I02 (0.9795)	DLS I03 (0.9000)
Resolution range (Å)	54–1.95 (2.06–1.95)*^b^*	51–2.19 (2.25–2.19)	66–2.8 (2.95–2.80)	48–2.30 (2.36–2.30)
Multiplicity	2.3 (2.2)	5.4 (2.8)	3.2 (3.0)	3.3 (2.6)
Significance (〈*I*〉/σ(*I*))	8.7 (3.6)	15 (2.4)	5.4 (2.3)	15.8 (2.2)
No. of unique reflections	32,357	11,235	16,258	18,488
Completeness (%)	96.9 (97.3)	97.1 (77.8)	98.0 (98.2)	99.2 (94.3)
*R*_merge_ (%)*^c^*	8.8 (41.2)	7.2 (49.7)	12.4 (37.4)	4.3 (36.6)

**Refinement statistics**				
*R*_cryst_	22.5	21.7	24.6	22.6
*R*_free_	25.3	25.6	29.1	25.5
Non-hydrogen atoms				
All	2,941	1,720	5,214	3,022
Water	141	30	16	26
Mean overall B (Å^2^)	24.7	40.4	50.9	46.6

**Root mean square deviations from ideal values**				
Bond distance ( Å)	0.010	0.006	0.003	0.002
Bond angle (degrees)	1.2	0.91	0.747	1.2

*^a^* Diamond light source.

*^b^* Values in parentheses refer to the outer resolution shell.

*^c^ R*_merge_ = Σ*_hkl_*Σ_sym_|*I* − 〈*I*〉|/Σ*_hkl_I*.

For the NCS-1·D2R complex, molecular replacement used the structure of human NCS-1 (human and rat NCS-1 have 99% sequence identity; PDB accession 1G8I) and PHASER (31), as implemented from within PHENIX (32). The results gave a clear solution to the rotation (*Z* score 7.2) and translation functions, with one NCS-1 molecule in the asymmetric unit. This was followed by automated model building and refinement in PHENIX. At this stage, electron density for the helix for chain B was readily apparent, and a model for the helix was built in manually using COOT (33). Further refinement using REFMAC 5.6 (34) from the CCP4 suite (35) revealed density for a second copy of the bound peptide, also as a helix, located adjacent to chain B. This was built manually as chain C. Final rounds of refinement were carried out assisted by the PDB_REDO server (36), accompanied by minor manual rebuilding. The final structure was complete from Leu-10 to Val-190 and contained three Ca^2+^ ions and a single potassium ion.

Data for the uncomplexed NCS-1 were processed using MOSFLM (37) and SCALA (30). The structure was determined using the NCS-1 coordinates from the NCS-1·D2 peptide complex and the program MOLREP (38), as implemented from within the CCP4 program suite (39). The molecular replacement procedure revealed a dimer in the asymmetric unit, and an initial model for both chains was built using BUCCANEER, within the CCP4 program suite (39). This was followed by manual rebuilding using COOT (33), and refinement was carried out using REFMAC (34). The final model contained three Ca^2+^ ions per chain, as for the NCS-1·peptide complex, plus two ions that bridged crystal contacts in the structure, and were built as Na^+^, presumably acquired from the crystallization buffer. Electron density was weak or missing for residues 1, 51–59, 132–138, and 190 (chain A) and 1–9, 134–138, and 186–190 (chain B).

The structure of the NCS-1·GRK1 complex was determined using the coordinates from the A chain of the NCS-1 apoprotein and the program MOLREP (38), as implemented from within the CCP4 program suite (39). The molecular replacement procedure revealed a dimer in the asymmetric unit; minor manual rebuilding was carried out using COOT (33) and initial refinement with REFMAC 5.6 (34). Electron density for a single GRK1 peptide per NCS-1 monomer, in a helical conformation, was readily detectable. Final rounds of refinement were carried out assisted by the PDB_REDO server (36), accompanied by minor manual rebuilding. The final model contained one GRK1 peptide and three Ca^2+^ ions per chain. Electron density was weak or missing for residues 1–7, 134–137, and 185–190 (chain A) and for 1–7, 133–138, and 185–190 (chain B).

Data for NCS-1ΔCT were processed using MOSFLM (37) and SCALA (30). Molecular replacement was in PHASER (31) using as search model the structure of the NCS-1·D2R complex where the binding peptide had been removed and the C terminus truncated to match construct composition. The four molecular copies of NCS-1ΔCT in this crystal form were readily identified in this way. Manual model rebuilding was in COOT (33) and refinement used PHENIX 1.9 (32) applying NCS restraints across the four molecular copies in the asymmetric unit and TLS refinement (one group per molecular copy). As for the NCS-1·peptide complexes, the final NCS-1ΔCT model contained three Ca^2+^ ions per chain. Electron density was weak or missing for the following residues that are not included in the model: 1–15, 136–143, and 175–177 (chain A); 1–7, 138–142, and 176–177 (chain B); 1–7, 36–42, 139–142, and 175–177 (chain C); and 1–7, 40–42, and 174–177 (chain D). Data statistics and model parameters are listed in Table 1. Protein-protein contacts were analyzed using PISA (40) and PIC (41).

## Results

### 

#### 

##### Structure of Uncomplexed Rat NCS-1

**FIGURE 1.**
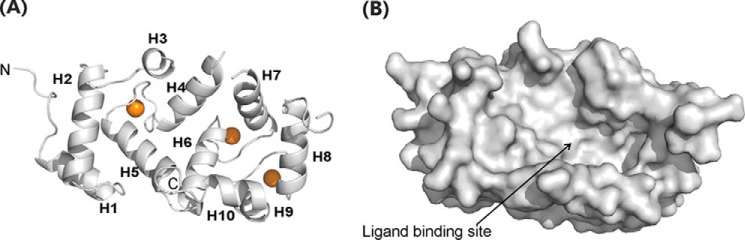
**Structure of rat Ca^2+^/NCS-1 apoprotein (PDB code 5AEQ).** *A,* backbone schematic of NCS-1 apoprotein with α-helices 1–10 labeled. Ca^2+^ ions are shown as *brown spheres. B,* surface representation for NCS-1 showing the large solvent-exposed hydrophobic crevice.

**FIGURE 2.**
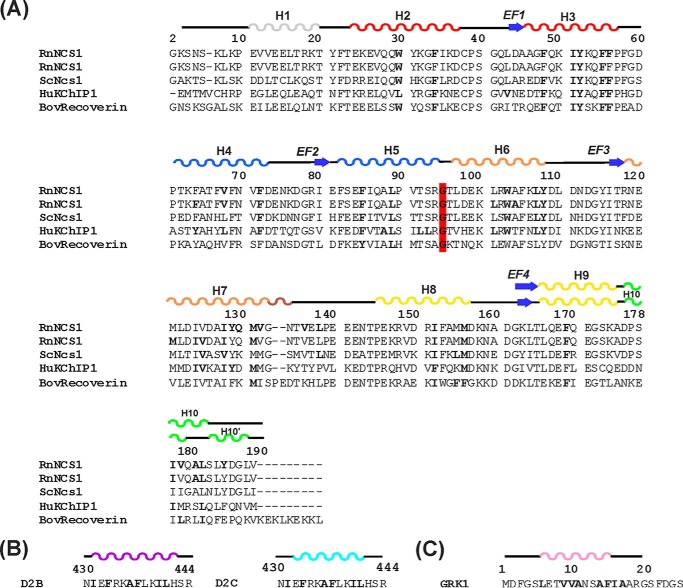
**Summary of interaction sites of NCS proteins.** *A,* alignment of the primary sequence of rat NCS-1 (RnNCS-1; Swiss-Prot P62168) with *Saccharomyces cerevisiae* Ncs1 (ScNcs1; Swiss-Prot Q06389), human KChIP1 (HuKChIP1; Swiss-Prot Q9NZI2.2) and bovine recoverin (BovRecoverin; Swiss-Prot P21547). RnNCS-1 is repeated to separately identify NCS-1 residues that form intermolecular interactions with D2R (*top sequence*) and GRK1 (*2nd sequence*) peptides. Numbering of amino acids is in accordance to the primary sequence of rat NCS-1. Secondary structure elements are derived from the structures of rat NCS-1 in complex with D2 receptor peptide (PDB 5AER). The C-terminal region forms helix 10 in the NCS-1 apoprotein and in the NCS-1·GRK1 complex (denoted by the *upper line* of secondary structure for this region). In the D2R peptide complex, helix 10 is replaced by a helix-turn-helix, and this is denoted as helices 10 and 10′. The four EF-hands, EF1, EF2, EF3, and EF4, are colored *red, blue, orange* and *yellow*, respectively. The short 3_10_ helix between EF3 and EF4 is colored *brown* and the C-terminal region in *green*. The hydrophobic residues that form interactions in the different complexes are highlighted in *boldface*; the different complexes are NCS-1·D2R complex (PDB code 5AER), the RnNCS-1·GRK1 complex, ScNcs1·Pik1 (PDB Code 2JU0), HuKChIP1·Kv4.3 (PDB Codes 2NZ0 and 2I2R) and BovRecoverin/rhodopsin kinase (PDB code 2I94). The conserved Gly-95 that divides NCS proteins into the N- and C-lobes is highlighted in *red*. The hydrophobic contacts are analyzed from the deposited structures using the Protein Interactions Calculator Webserver (38). *B* and *C,* amino acid sequence of D2 dopamine receptor (*B*) and GRK1 (*C*) peptides with residues involved in hydrophobic interactions with NCS-1 in *boldface* and the α-helices colored in *magenta/cyan* and *pink*, respectively, in the D2R and GRK1 complex. In the NCS-1·GRK1 peptide structure, a hydrophobic triad is formed between Met-156, Ile-179, and the peptide residue Ile-16.

The crystal structure of rat NCS-1 (hereafter referred to simply as NCS-1) includes four canonical EF- hand folds (Fig. 1*A*); the overall fold is similar to that adopted by human Ca^2+^/NCS-1 (27) and its yeast ortholog frequenin (Fig. 2*A*) (42). Electron density consistent with three Ca^2+^ ions in EF2, EF3, and EF4 was observed; as expected for the NCS family of proteins, EF1 does not bind Ca^2+^ (5). EF1 and EF2 form the N-lobe and EF3 and EF4 the C-lobe; intra-lobe hydrophobic and hydrogen bond interactions help to stabilize the conformation of the two lobes. The inter-helix angles of the EF1–4 are, respectively, 91, 96, 90, and 105°; these are similar to the inter-helix angles found for Ca^2+^-bound EF hands in other proteins, for example Ca^2+^/calmodoulin (CaM) and KChIP1 (43). Structurally, both the N- and C-lobes overlay well with each other, with an overall root mean square deviation of 1.187 Å for backbone atoms. Residues Pro-177–Leu-183 form the final helix 10 (H10), which makes several hydrophobic contacts with the C-lobe as follows: Val-180 → Leu-97, Leu-183 → Leu-97, Leu-183 → Leu-101, Ile-179 → Leu-101, and Ile-179 → Met-156. The N- and C-lobes are connected by Gly-95, which is highly conserved in the NCS protein family but is not found in CaM (Fig. 2*A*). Consequently, in the Ca^2+^-bound state, NCS-1 folds in such a way that the two lobes are oriented to jointly present a large, solvent-exposed, concave hydrophobic crevice onto which peptide, proteins, and other hydrophobic molecules can dock and bind (Fig. 1*B*).

**FIGURE 3.**
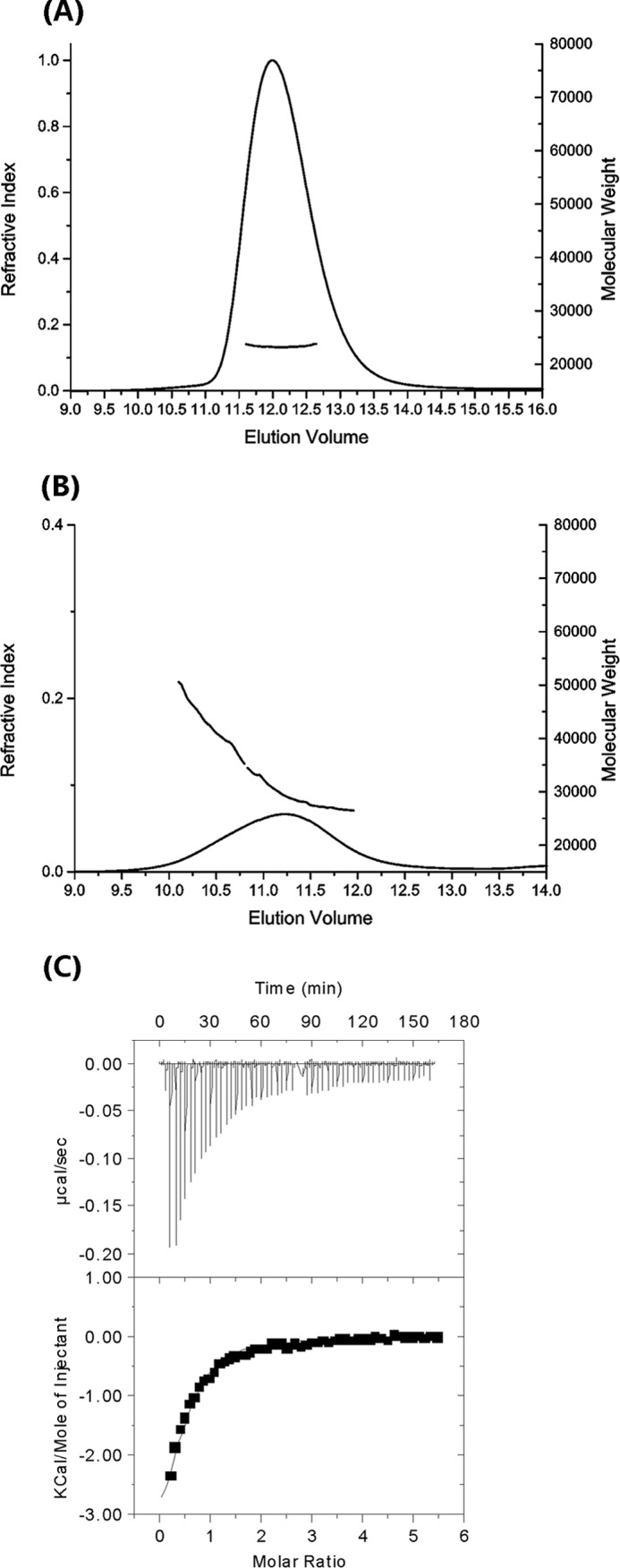
**MALLS and ITC analyses.** *A* and *B,* MALLS data for NCS-1, showing a peak consistent with a monodisperse sample and an expected molecular mass of 21,880 ± 200 daltons. The average molecular mass per volume unit is shown in *gray* and the differential refractive index in *black. B,* MALLS data for Ca^2+^/NCS-1(1–177) (NCS-1ΔCT), the protein elutes as a polydisperse peak with molecular mass ranging from 29,300 to 42,000 daltons. Other details are as for *A. C,* ITC titration data for the binding of the D2R peptide to NCS-1; data from two runs were concatenated to achieve a saturating isotherm. The data were fitted to a two-site binding model to yield *K_d_* values of 40 ± 6 μm.

The crystal structure contained two copies of NCS-1 in the asymmetric unit, related by noncrystallographic 2-fold symmetry. However, analysis of rat NCS-1 by SEC MALLS (Fig. 3*A*) shows that NCS-1 is monomeric in solution with an estimated molecular mass of 21,880 ± 200 daltons.

##### Isothermal Titration Calorimetry

The region of the D2R recognized by NCS-1 was narrowed down to the peptide sequence TFNIEFRKAFLKILHC (18). A crystal structure of the homologous D3 receptor has since become available (44), which places this sequence at the end of the 7th transmembrane helix, with the TF dipeptide forming the last two residues of this helix. We found that the peptide TFNIEFRKAFLKILHC was insoluble in water, but removal of the first two N-terminal amino acids improved solubility. To overcome potential disulfide bridge formation, the C-terminal Cys was replaced by a Ser, and an additional Arg was added to further improve solubility. Hence, the peptide NIEFRKAFLKILHSR was used for the studies described here and is referred to as the D2R peptide. Because of limited solubility, the isothermal titration calorimetry experiment was performed by titrating NCS-1 into the D2R peptide. The binding isotherm was fitted to two models. A sequential binding model gives *K_d_* values of ∼43 and 58 μm, with very similar Δ*H* and Δ*S* values for the two interactions. A two-site nonsequential model with a fixed stoichiometry of 1:2 NCS-1·peptide gives a *K_d_* of 40 ± 6 μm for both sites (Fig. 3*C*). Because there is no other evidence to suggest a sequential binding mode, we have selected the simplest two-site binding mode for the interpretation of these data, which shows that the interactions are entropically driven, with Δ*H* = −2.06 ± 0.07 kcal/mol and −*T*Δ*S* = −3.9 ± 0.8 kcal/mol. The favorable entropy is expected, given the expected hydrophobic nature of the interactions between the peptide and the protein. The binding isotherm is typical of a weak interaction; it is possible to derive reliable dissociation constants because a sufficiently large proportion of the binding isotherm was used for the analysis (45). The synthetic peptide from GRK1, RK25, is the same peptide sequence that was used to form a complex with recoverin (46). This peptide had limited solubility and a propensity to aggregate, making it difficult to reliably measure its binding affinity with NCS-1 using either the ITC or an NMR method.

##### Structure of the Ca^2+^/NCS-1·D2R Peptide Complex

**FIGURE 4.**
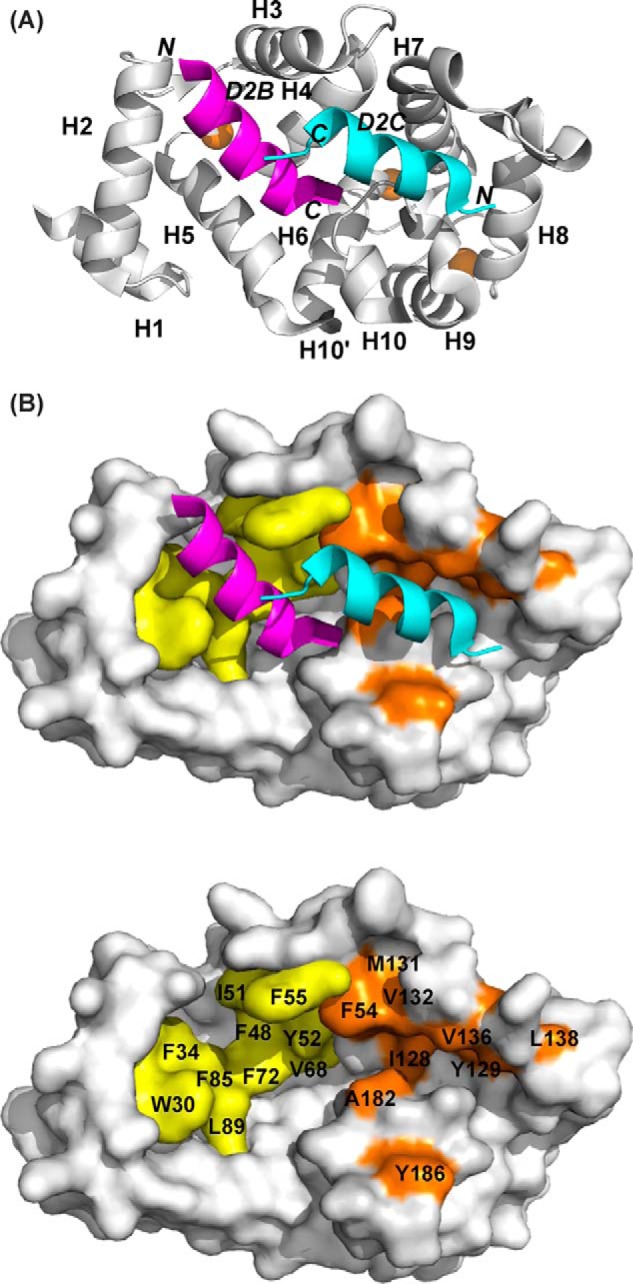
**Structure of rat Ca^2+^/NCS-1 in complex with D2R peptide (PDB code 5AER).** *A,* backbone schematic representation of the NCS-1 in complex with D2R peptide, viewed from the binding interface, with α-helices 1–10 indicated. Two D2R peptides bind independently, one at the N-lobe site (D2B, *magenta*) and the other at the C-lobe site (D2C, *cyan*). *N* and *C* are the N and C termini of the D2R peptide. Ca^2+^ ions are shown in *brown. B, top panel,* molecular surface of NCS-1 showing the hydrophobic residues involved in D2R binding in the N-lobe site in *yellow* and the C-lobe site in *brown. Bottom panel,* same as *top panel* but with bound peptides removed and key interacting residues labeled.

Diffraction data were collected from crystals of rat NCS-1 in complex with the D2R peptide, and the structure was determined to 2.19-Å resolution. The complex adopted a different crystal form from the NCS-1 structure without peptide bound, with one NCS-1 chain in the asymmetric unit. The most striking feature is that NCS-1 binds two copies of the D2R, one in the N-lobe site (referred to as peptide D2B) and the other in the C-lobe site (peptide D2C) (Fig. 4*A*). Both peptides bind as amphipathic helices, with D2B forming a slightly longer helix than D2C. The two peptides are bound with their C termini pointing toward the center of the NCS-1 molecule. EF1/EF2 residues form most of the interactions with D2B and EF3/EF4 with D2C (Fig. 4*B*), although there are some exceptions. In addition, the C-terminal region Ile-179–Val-190 is also involved in interactions with D2C. The main interactions between the peptides and NCS-1 in the two binding sites are summarized in Table 2.

**TABLE 2:**
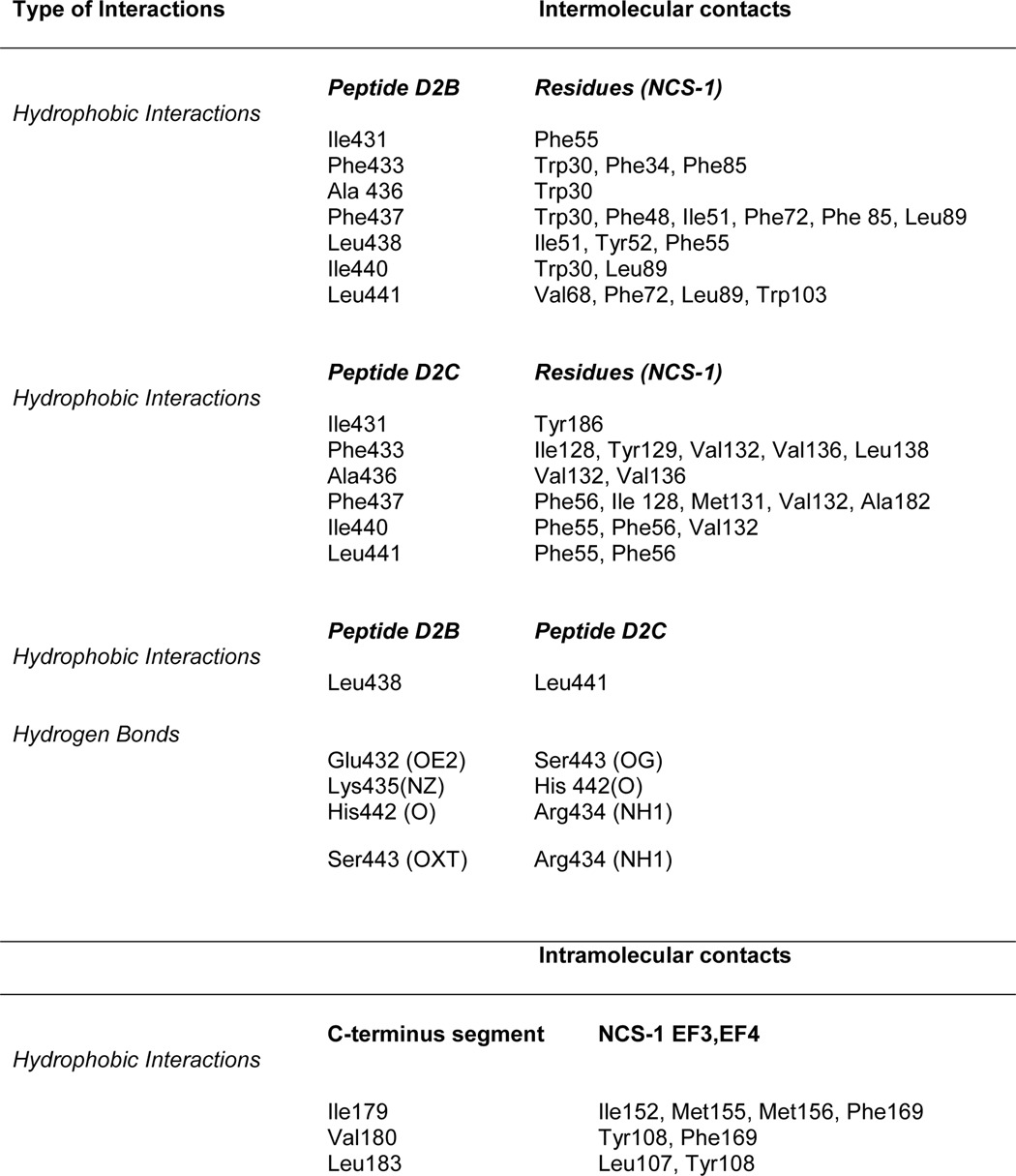
**Main intermolecular and intramolecular interactions involving D2 receptor peptides and NCS-1**

**FIGURE 5.**
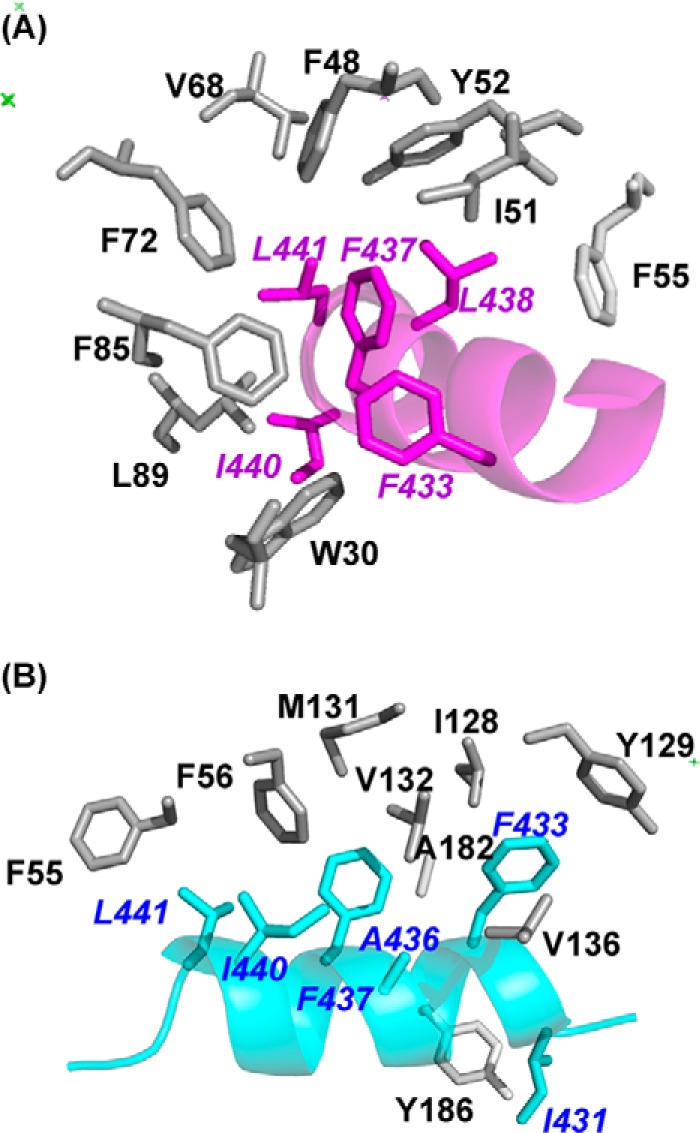
**Intermolecular interactions between rat Ca^2+^/NCS-1 and D2R peptide.** *A,* expanded view of the hydrophobic interactions between the side chains of D2B (*magenta*) and the N-lobe. *B,* detailed view of the hydrophobic interactions between the side chains of D2C (*cyan*) and the C-lobe. Peptide residue labels are in *italics*.

In the N-lobe site, the protein-peptide interface is formed by 11 hydrophobic residues from NCS-1 and 7 residues from D2B. The total buried surface areas of 737 Å^2^ for NCS-1 and 814 Å^2^ for the peptide are slightly higher than for other protein·peptide complexes (47). The loop comprising residues Gln-54–Gly-59 was omitted from the structure of Ca^2+^/NCS-1 alone, but electron density was observed when the D2R peptides were present. Peptide residues Ile-431, Phe-433, Ala-436, Phe-437, Leu-438, Ile-440, and Leu-441, which are completely conserved between the D2 and D3 dopamine receptors and 50% conserved in the D4 receptor, make hydrophobic contacts with the conserved NCS-1 residues Trp-30, Phe-34, Phe-48, Ile-51, Tyr-52, Phe-55, Val-68, Phe-72, Phe-85, Leu-89, and Trp-103 (Table 2 and Fig. 5*A*). The non-interface residues of the D2B helix, consisting of the positively charged and polar residues Glu-432, Lys-435, Lys-439, His-442, and Ser-443, point toward the solvent. Phe-437 and Leu-441 make the most contacts with the protein, with their side chains completely buried at the hydrophobic interface. More interactions are formed with Trp-30 of NCS-1 than any other residues in this pocket, with Phe-55 being the most buried at the protein-peptide interface. Attempts to test the importance of Trp-30 were hampered by the severe aggregation of the W30L mutant protein.

Binding of D2C at the C-lobe site gives rise to several significant conformational changes in NCS-1. The buried surface areas for NCS-1 and the D2 receptor peptide are, respectively, 600 and 681 Å^2^, both notably lower than the buried surface for D2B binding; this is explained by D2C making fewer intermolecular contacts than D2B (Table 2). Hydrogen bonds between Gln-181 of NCS-1 and Asn-430 of D2C orient the peptide such that it binds in the opposite direction to D2B (Fig. 4*A*).

**FIGURE 6.**
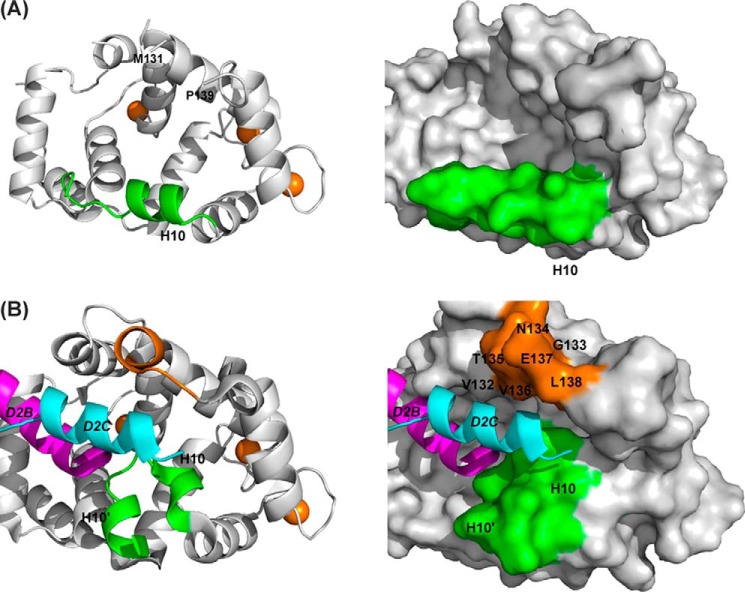
**Comparison of the conformation of the C-lobe in unliganded rat Ca^2+^/NCS-1 and the NCS-1·D2R complex.** *A,* backbone schematic (*left*) and molecular surface representation (*right*) of the C-lobe of unliganded NCS-1 (PDB code 5AEQ). The C-terminal helix 10 is colored *green,* and Ca^2+^ ions are shown in *brown*. Electron density for Val-132–Leu-138 was weak, and these residues were therefore omitted from the model. *B,* backbone schematic and molecular surface representations of the C-lobe of NCS-1·D2R complex (PDB code 5AER). The C-terminal helix-loop-helix (helices 10 and 10′) formed by residues Pro-177–Leu-189 is colored *green*. The D2B peptide in the N-site is colored *magenta*; the D2C peptide in the C-site is colored *cyan,* and residues Val-132–Leu-138 are colored *brown*.

The main structural changes that occur on binding are found in the EF3-EF4 linker region (Val-132–Pro-139) and in the C-terminal segment (Asp-176–Val-190). In the uncomplexed protein, electron densities for Val-132–Leu-138 are missing, implying that this region is unstructured (Fig. 6*A*); in the presence of D2C, the region immediately after EF3 adopts a short 3_10_ helix (Gly-133 to Thr-135), and electron density for the loop is now defined (Fig. 6*B*). This induced-fit structural stabilization effectively increases the height of one side of the binding crevice (Fig. 6*B*, *right*). The interactions between residues Ile-128, Tyr-129, Val-132, Val-136, and Leu-138 from this region and the hydrophobic side of the D2C amphipathic helix form the predominant contacts between the peptide and the protein (Fig. 5*B*). Additional hydrophobic contacts occur between D2C, including those with Phe-55 and Ala-182 and with Tyr-186 in the C-terminal segment (Fig. 5*B* and Table 2). It is interesting to note that the EF3/EF4 linker is the least conserved segment in the NCS family of proteins (Fig. 2), and the residues that are stabilized upon D2R peptide binding are, therefore, likely to have a role in determining the specificity of the different NCS family members.

In the absence of D2C, the C-terminal segment residue Pro-177–Leu-183 forms α-helix 10 (H10) (Fig. 6*A*), which makes hydrophobic intramolecular side-chain interactions, hydrogen bonds, and ionic contacts with helices 5, 6, and 8. Helix 10 forms one edge of the large ligand-binding hydrophobic crevice, rather than occupying the hydrophobic ligand-binding crevice. When D2C is bound, the C-terminal region switches to a “U” shaped conformation comprising two antiparallel 3_10_ helices, Pro-177–Val-180 and Ser-184 to Gly-188, connected by a turn made up of residues Gln-181–Leu-183 (Fig. 6*B*). The C-terminal segment forms many intramolecular side-chain interactions with the hydrophobic binding groove as follows: Ile-179 → Ile-152, Met-155, Met-156, Phe-169; Val-180 → Tyr-108, Phe-169, and Leu-183 → Leu-107, Tyr-108 (Table 2). Furthermore, extensive hydrophobic side-chain interactions within the helix-turn-helix motif involving residues Val-180, Ala-182, Leu-183, Leu-185, Tyr-186, and Leu-189 serve to maintain and stabilize this conformation.

In summary, two molecules of the D2R peptides bind in the hydrophobic crevice of NCS-1, with the most significant conformational changes observed in the EF3/EF4 linker and the C-terminal region, which adopts a helix-loop-helix conformation in the complex.

##### Structure of Ca^2+^/NCS-1·GRK1 Peptide Complex

**FIGURE 7.**
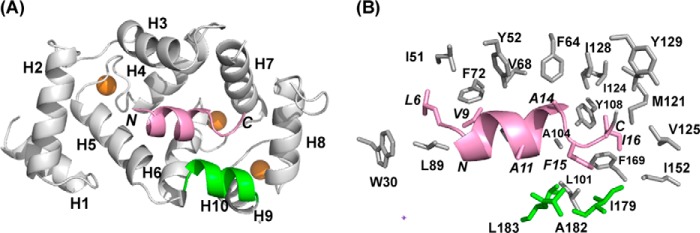
**Structure of rat Ca^2+^/NCS-1 in complex with GRK1 peptide (PDB code 5AFP).** *A,* backbone schematic representation of the NCS-1 in complex with GRK1 peptide, viewed from the binding interface, with α-helices 1–10 indicated. The GRK1 peptide (colored *pink*) is bound in the hydrophobic crevice. *N* and *C* label the N and C termini of the GRK1 peptide. The NCS-1 C-terminal helix 10 is colored *green*. Ca^2+^ ions are shown as *brown spheres. B,* detailed view of the hydrophobic interactions between the side chains of GRK1 peptide (*pink*) and NCS-1. Peptide residues are in *italics*; residues from the C-terminal region are colored *green*.

Determination of the crystal structure of the complex of Ca^2+^/NCS-1 with the GRK1 peptide revealed a different mode of recognition compared with D2R. Only one molecule of the GRK1 peptide is bound, deep into the hydrophobic groove of NCS-1 (Fig. 7*A*). The GRK1 peptide binds as a two-turn α-helix involving residues Ser-5–Ala-14, with the peptide aligned parallel with the N-C axis of NCS-1; electron density for the remainder of the peptide was not observed. The contacts made between the protein and peptide are summarized in Table 3. The protein-peptide interface is formed by 29 residues from the protein and 8 hydrophobic residues from GRK1 peptide, with total buried surface areas of 711 Å^2^ for NCS-1 and 921 Å^2^ for the peptide. The electron densities for Gln-54–Gly-59 and part of the EF3/EF4 linker region are missing. The main protein-peptide contacts are formed by residues Leu-6, Val-9, Val-10, Ala-14, Phe-15, Ile-16, and Ala-17 of GRK1 peptide (Fig. 7*B*). These residues fit into the hydrophobic crevice, with side-chain hydrophobic contacts formed between them and the exposed highly conserved hydrophobic residues of NCS-1 from both the N-lobe, C-lobe, and C-terminal helix 10 (Fig. 7*B* and Table 3).

**TABLE 3:**
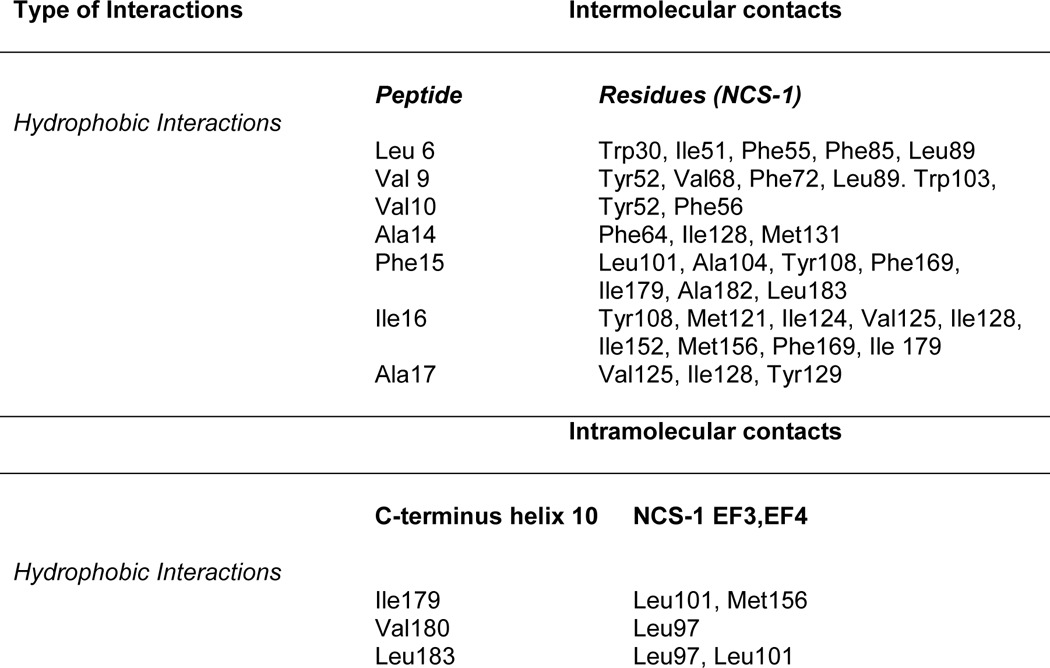
**Main intermolecular and intramolecular interactions involving GRK1 peptide and NCS-1**

**FIGURE 8.**
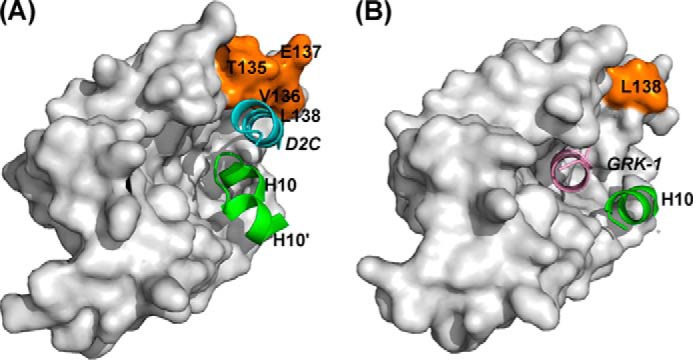
**Comparison of D2R and GRK1 peptide binding to rat Ca^2+^/NCS-1.** *A,* view from the N-terminal end of the NCS-1·D2R peptide complex (PDB code 5AER) showing the location of the D2R peptide that is bound in the C-lobe of NCS-1 (denoted by *D2C*). For clarity, the D2B peptide in the N-lobe site is omitted. The D2C peptide is colored *cyan*; residues from the EF3/EF4 linker region (Val-132–Leu-138) are colored *brown,* and the C-terminal region comprising helices 10 and 10′ is shown in *green. B,* view from the N-terminal end of NCS-1 bound to GRK1 (PDB code 5AFP). The GRK1 peptide is colored *pink*, residue Leu-138 from the EF3/EF4 linker region is colored *brown,* and the C-terminal region comprising helix 10 is shown in *green*.

The biggest differences between the GRK1 peptide and D2R peptide complexes lie in the conformations of the C-terminal region of NCS-1 and the EF3/EF4 linker region. In the D2R peptide complex, residues Pro-177–Val-190 form a 3_10_-helix-loop-3_10_-helix and share the C-lobe-binding site with one of the D2R peptides (Fig. 8*A*). Rather than being buried deep into the hydrophobic crevice, this D2R peptide is displaced to one side of the binding site (Fig. 8*A*) with hydrophobic interactions between the peptide and the EF3/EF4 linker region stabilizing the structure of the complex. In contrast, the C-terminal region of NCS-1 in the NCS-1·GRK1 peptide complex forms an α-helix that lies to one side of the hydrophobic crevice. The electron densities for Asn-134–Glu-137 of the EF3/EF4 linker region are missing, making the C-lobe site very solvent-exposed and allowing the GRK1 peptide to bind deep into the hydrophobic pocket (Fig. 8*B*). Interestingly, the NMR structure of yeast Ncs1 bound to the N terminus of Pik1 also shows the C-terminal segment of the Pik1 peptide located deep in the hydrophobic crevice (48), although in this case, the C-terminal region of Ncs1 was not be defined due to conformational exchange.

##### Conformation of the C-terminal Region of Rat Ca^2+^/NCS-1

**FIGURE 9.**
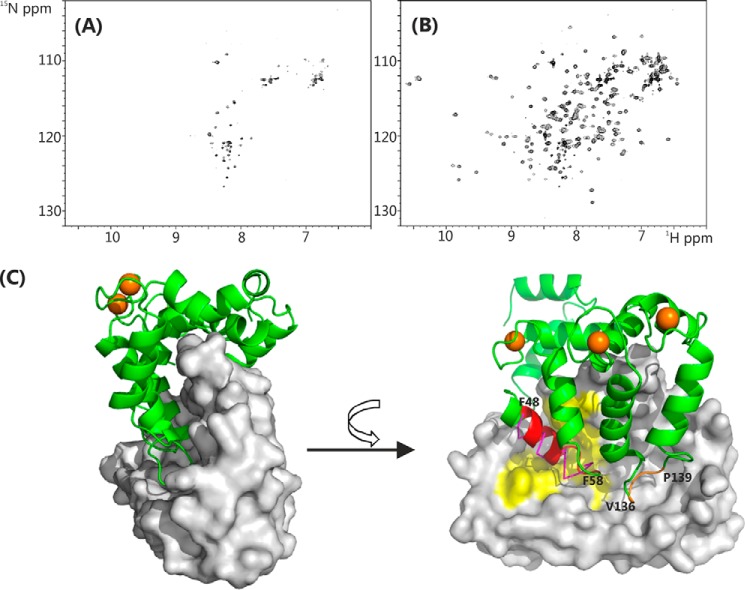
**NMR spectra and structure of rat Ca^2+^/NCS-1ΔCT (PDB code 4YRU).** *A,*^1^H-^15^N heteronuclear single quantum coherence spectra of ^15^N Ca^2+^/NCS-1ΔCT; *B,* in the presence of 5-fold excess of D2R peptide in 50 mm Tris-HCl buffer, 50 mm NaCl, 5 mm CaCl_2_, pH 6.5, 298 K. *C,* structure of the dimer of NCS-1ΔCT. *Left,* one monomer is shown as a backbone schematic and the other as a surface representation. *Right,* view of the hydrophobic binding site. Residues from helix 3 and the EF1/EF2 loop (Phe-48–Phe-58) are colored *red,* and residues in the EF3/EF4 loop region (Val-136–Pro-139) are colored *brown*. Conserved hydrophobic surface-exposed residues in the N-lobe site, which are involved with binding the D2R peptide, are highlighted in *yellow* (similar to those highlighted in Fig. 4*B*). D2R peptide in the N-site is shown with *magenta* ribbon for comparison. Ca^2+^ ions are shown as *brown spheres*.

The D2R and GRK1 complex structures provide strong evidence for the importance of the C-terminal region of NCS-1 in peptide recognition. To investigate this phenomenon further, residues Ser-178–Val-190 were deleted to create a C-terminal truncated mutant, NCS-1ΔCT. The solution behavior of NCS-1ΔCT was significantly different from that of the full-length protein. Size exclusion chromatography-multiangle laser light scattering analysis showed that NCS-1ΔCT was polydisperse, with a molecular mass between 29,300 and 42,000 daltons, compared with that for the monodisperse full-length protein of 21,880 daltons (Fig. 3*B*). The polydispersity was confirmed in the NMR spectra of NCS-1ΔCT, which showed significant concentration-dependent line broadening (Fig. 9*A*), similar to that previously reported (49). Titration with the D2R peptide improved the line widths and overall spectral quality (Fig. 9*B*). We infer from these results that without the C-terminal region the NCS-1 hydrophobic binding site is completely exposed, causing the protein to self-associate.

NCS-1ΔCT crystallized as a symmetrical homodimer with the structure resembling a pair of clasped hands, with the “fingers” of one hand inserting into the “palm” of the other. The palm is the hydrophobic ligand-binding crevice, and the fingers are formed by hydrophobic residues positioned on the surface of the one subunit in such a way that they slot snugly into the hydrophobic crevice of the other subunit (Fig. 9*B*). Hydrophobic residues from helix 3 and the EF1/EF2 loop (residues Phe-49–Phe-58) bind to the N-lobe site; residues Val-136–Pro-139 in the EF3/EF4 loop region bind to the C-lobe site (Fig. 9*B*). Interestingly, these two loop regions have been singled out as the ones that are poorly defined in the unliganded protein (see above). The protein-protein interface buries a large area of about 1854 Å, which means that nearly one-third of the surface of each subunit forms the interface in the homodimer. Similar C-terminal truncations of NCS-1 (49) and recoverin (50) have been studied previously for ligand binding activities, although no high resolution structural characterization was performed.

In the NMR solution studies, we and others (23, 49) have reported that the C-terminal region (Ile-179–Val-190), in either the unliganded or complexed protein, exists in intermediate chemical exchange on the NMR time scale between several conformations, precluding the determination of the precise structure of this region of the protein. At first glance, the mobility of the C-terminal region observed in the NMR experiments appears to contradict the observation of a single conformational state in the crystal structure of the full-length protein, where this region forms a well defined helix at the edge of the hydrophobic crevice. However, it is likely that this conformation is one of many structures adopted by the C-terminal region in solution.

## Discussion

The structural basis for the different physiological roles of the many neuronal calcium sensor protein interactions remains unclear, as there are only a limited number of relevant high resolution complex structures (6). The identification of the key determinants of binding specificity (51) and the way diversity is conferred remain outstanding questions. With the high homology of the primary polypeptide sequences and conservation of the NCS protein folds, it is not immediately apparent why and how these NCS proteins show specificity and diversity.

The four crystal structures of the two NCS-1 complexes, the unliganded and truncated Ca^2+^-bound proteins, highlight the following significant features. (i) Two molecules of D2R peptide bind to NCS-1, whereas only one molecule of the GRK1 peptide is found in the hydrophobic binding groove. (ii) The conformation of the N-lobe site appears to be conserved across the two NCS-1 complexes; in contrast, the C-lobe site configuration is variable, due to the different conformations adopted by the EF3-EF4 linker region and the C-terminal residues Pro-177–Val-190. (iii) The C-terminal region partially occupies the C-lobe site in the D2R peptide complex, providing interaction sites for the peptide. (iv) Removal of the C-terminal segment (Ser-178–Val-190) leads to a dimerization of NCS-1.

The important hydrophobic residues in NCS-1 protein, which form the N-lobe ligand binding pocket, include the highly conserved residues Trp-30, Phe-34, Phe-48, Ile-51, Tyr-52, Phe-55, Phe-85, and Leu-89 (Fig. 2 and Tables 2 and 3); in all the other complexes studied, these residues have been shown to be important for binding the target peptides and proteins (Fig. 2). In support of this, the functional importance of certain hydrophobic residues within the hydrophobic crevice of NCS-1, including Leu-89, have been demonstrated in an *in vivo* rescue study in *Caenorhabditis elegans* (52). With the exception of the stabilization of the EF1/EF2 linker Gln-54–Gly-59 in the D2R peptide complex, binding of both the D2R and GRK1 peptide induces minimal conformational changes to the N-lobe of the NCS-1 protein. In contrast, more substantial structural changes occur at the C-lobe, particularly in the region Val-132–Pro-139 within the poorly conserved EF3-EF4 linker and the C-terminal (Pro-177–Val-190) region; the latter adopts a 3_10_-helix-loop-3_10_-helix in the D2R peptide complex and an α-helix in the GRK1 peptide complex.

**FIGURE 10.**
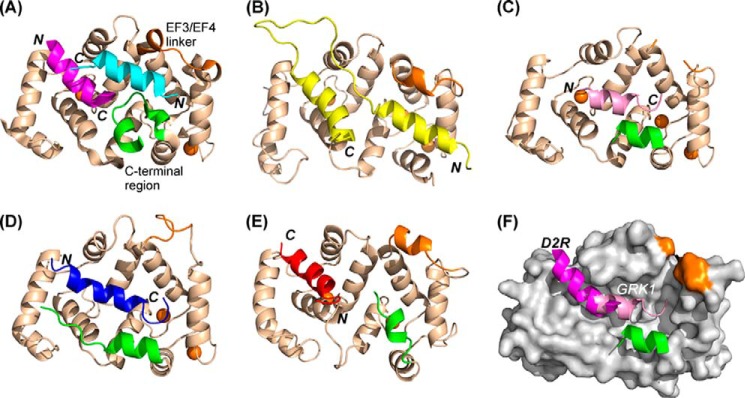
**Comparison of the structures of NCS protein complexes.** Schematic representations are shown of the structures of NCS-1 in complex with two molecules of D2R peptide (*magenta* and *cyan*) (PDB 5AER) (*A*), ScNcs1in complex with fragment of Pik1 (*yellow*) (PDB 2JU0) (45) (*B*), NCS-1 in complex with one molecule of GRK1 peptide (*pink*) (PDB 5AFP) (*C*), and KChIP1 with a fragment of bound Kv4.3 (*blue*) (PDB 2I2R) (50) (*D*). *E,* recoverin bound to the N terminus of GRK1 residues 1–25 with GRK1 peptide (*red*; PDB 2I94) (52). *F,* overlay of structures 5AER and 5AFP showing the locations of the D2R bound in the N-site and GRK1 peptides. The peptide orientations are indicated as *N* and *C* in *bold italics,* and the orientations of the NCS protein are identical in all the structures. The EF3/EF4 linker is colored *brown* and the C-terminal region *green*; for clarity these regions are indicated only for the NCS-1·D2R peptide complex. In all the structures, Ca^2+^ ions are shown as *brown spheres*.

A comparison of the NCS-1·D2R and NCS-1·GRK1 complex structures reveals some interesting features (Fig. 10). The NCS-1·D2R complex (Fig. 10*A*) closely resembles the NMR structure of the yeast NCS-1 homolog, frequenin, in complex with yeast Pik1 (Fig. 10*B*) (48), where two helical regions from the Pik1 polypeptide(119–256) bind separately to the N-lobe and C-lobe hydrophobic pockets (48); the C terminus in this structure (PDB 2JU0) is undefined.

Interestingly, the NCS-1·GRK1 complex resembles the KChIP1·Kv4.3 complex (PDB 2NZ0 and 2I2R), with the bound peptides forming an α-helix that spans both the N- and C-lobe sites (Fig. 10, *C* and *D*) (53, 54). The C-terminal α-helix in both complexes form one edge of the hydrophobic groove. In both the NCS-1·GRK1 peptide and KChIP1·Kv4.3 complexes, hydrophobic side-chain interactions are found between the C-terminal region and the bound peptide, indicating that that region helps to stabilize the binding of the partner peptides. Somewhat surprising are the differences observed between the NCS-1·GRK1 peptide complex and the NMR structure of the same peptide in complex with its *in vivo*-characterized NCS partner, recoverin (PDB 2I94) (Fig. 10*E*) (46), which shows the helical peptide binding only in the N-lobe site and in the opposite orientation to that found in the NCS-1·GRK1 complex. The C-lobe site in the recoverin structure is occupied by the last short helix 10 of recoverin itself.

The structures determined here provide detailed insights into both specificity and promiscuity of NCS proteins. First, the same peptide from GRK1 appears to be able to bind to two NCS proteins, recoverin and NCS-1, using different modes (Fig. 10, *C* and *E*). Second, the same NCS protein can bind targets in different ways (Fig. 10, *A* and *C*). Although previously proposed and discussed (6, 51, 56), to the best of our knowledge there have been no side-by-side comparisons of the different complex NCS structures to date. The different binding modes show that the C terminus is important in driving target specificity. In addition, as shown by NMR studies (48, 49), the C terminus in solution appears to be dynamic, adopting multiple conformations. The results here demonstrate that the mobility and flexibility of the C-terminal region have a particular functional significance. Flexibility allows the C-terminal region to serve two purposes. First, it serves to occlude the hydrophobic binding crevice in the absence of a partner protein and to prevent protein self-association and nonspecific interactions. Second, it modulates and regulates binding by adopting conformations to suit the binding partner. An observation in support of a physiological role for the C terminus is the consequence of a mutation in NCS-1 found in a patient with autism spectrum disorder (57), which resulted in a change in NCS-1 membrane association dynamics, coupled to enhanced conformational dynamics of the C terminus of the protein (58).

The more ubiquitous Ca^2+^-binding protein CaM, with over 80 structures of complexes in the Protein Data Bank, is known to exhibit diverse binding modes that are functionally important (59). This is made possible predominantly by the conformational flexibility of the linker between the N- and C-lobes. NCS-1 is reported to bind to over 20 proteins (6), despite the fact that it does not have the same flexible linker found in Ca^2+^/CaM. Instead, other elements of the NCS-1 structure confer the diversity and define the specificity, and the structures reported here provide an explanation of how this is achieved. The C-terminal region, together with the less conserved region in the EF3/EF4 linker, has the flexibility and structural plasticity to form the precise conformation of the C-lobe site to accommodate the binding target; hence, these components, C-terminal region, EF3/EF4 linker, and the binding peptide, all act in concert to form a unique protein complex. This observation would also explain why the EF3/EF4 linker and the C-terminal regions of the NCS family have low sequence and length conservation, compared with the rest of the protein sequence. Because of the more constrained structure of the NCS proteins, however, NCS proteins are likely to have less binding diversity than CaM.

It is interesting to note that the average sizes of the NCS peptide ligands are around 16 amino acids, and the hydrophobic crevice can accommodate two peptides of this size. The binding modes observed here agree with the D2R being a dimer (based on the structure of the highly homologous D3R) (44) and GRK1 being a monomer (55). We previously produced a model of NCS-1 interacting with the D3 receptor dimer, and we concluded that a minor conformational change in the receptor is required for it to bind to a single molecule of NCS-1 (23). Interestingly, the D2R and GRK1 peptides are not identically located in the hydrophobic binding groove of NCS-1; hence, there is a possibility that NCS-1 (and perhaps other NCS proteins) can simultaneously bind to two targets. An NCS-1·D2R·GRK2 ternary complex has previously been detected (18), and the structures determined here provide an explanation as to how this might be achieved, by a molecule of NCS-1 binding only to one subunit of the D2R, leaving most of the C-lobe free for GRK1 to bind, as shown in Fig. 10*F*.

This work shows how NCS proteins differentially affect specific aspects of neuronal function through their interactions with different target proteins. Genetics studies have shown that NCS proteins do not entirely overlap in function, where the loss of one is not compensated by another. This lack of redundancy is possibly due to the specificities different NCS proteins have toward their targets. The C-terminal region appears to act as a gate of the large hydrophobic crevice, and, together with the flexible EF3/EF4 linker it defines an appropriate binding site that filters out interactions that cannot be accommodated at this site. Finally, the structures here also show how NCS-1 could act as a small scaffold by allowing two proteins to bind simultaneously.

## Footnotes

5 The abbreviations used are:

NCS: neuronal calcium sensor
CaM: calmodulin
D2R: D2 dopamine receptor
ITC: isothermal titration calorimetry
PDB: Protein Data Bank.
